# Identification of mutations in porcine *STAT5A* that contributes to the transcription of *CISH*

**DOI:** 10.3389/fvets.2022.1090833

**Published:** 2023-01-17

**Authors:** Diwen Yao, Dongchun Guo, Yingkun Zhang, Zhihua Chen, Xiaowen Gao, Guiling Xing, Xiuqin Yang, Xibiao Wang, Shengwei Di, Jiancheng Cai, Buyue Niu

**Affiliations:** ^1^College of Animal Science and Technology, Northeast Agricultural University, Harbin, China; ^2^State Key Laboratory of Veterinary Biotechnology, Harbin Veterinary Research Institute, Chinese Academy of Agricultural Sciences (CAAS), Harbin, China; ^3^Lanxi Breeding Farm, Lanxi, China

**Keywords:** porcine, CISH, STAT5A, polymorphism, piglet growth trait

## Abstract

Identification of causative genes or genetic variants associated with phenotype traits benefits the genetic improvement of animals. CISH plays a role in immunity and growth, however, the upstream transcriptional factors of porcine *CISH* and the genetic variations in these factors remain unclear. In this study, we firstly identified the minimal core promoter of porcine *CISH* and confirmed the existence of STATx binding sites. Overexpression and RT-qPCR demonstrated STAT5A increased *CISH* transcriptional activity (*P* < 0.01) and mRNA expression (*P* < 0.01), while GATA1 inhibited *CISH* transcriptional activity (*P* < 0.01) and the following mRNA expression (*P* < 0.05 or *P* < 0.01). Then, the putative functional genetic variations of porcine *STAT5A* were screened and a PCR-SSCP was established for genotype g.508A>C and g.566C>T. Population genetic analysis showed the A allele frequency of g.508A>C and C allele frequency of g.566C>T was 0.61 and 0.94 in Min pigs, respectively, while these two alleles were fixed in the Landrace population. Statistical analysis showed that Min piglets with CC genotype at g.566C>T or Hap1: AC had higher 28-day body weight, 35-day body weight, and ADG than TC or Hap3: CT animals (*P* < 0.05, *P* < 0.05). Further luciferase activity assay demonstrated that the activity of g.508A>C in the C allele was lower than the A allele (*P* < 0.05). Collectively, the present study demonstrated that STAT5A positively regulated porcine *CISH* transcription, and SNP g.566C>T in the *STAT5A* was associated with the Min piglet growth trait.

## 1. Introduction

The improvement of piglet health and weight especially before weaning benefits pig production. Selection breeding, which changes the genetic trends of livestock populations, has become an efficient method to improve porcine health and growth performance. Identification of candidate genes or causative genetic variants associated with animal phenotype is primarily involved in genetic-based breeding.

Cytokine inducible SH2-containing protein (CISH) is a member of the suppressor of cytokine signaling (SOCS) proteins that function in blood and immune cell development (1), cell growth, and metabolism (2–5). Initial studies of *CISH* knockout mice demonstrated its functions in the regulation of immunity and disease (6). Thereafter, genetic variations within *CISH* were proven to associate with multiple diseases in humans, including malaria (7), bacteremia (7), tuberculosis (7, 8), persistent hepatitis B (9), multiple organ dysfunction syndrome (MODS) (10) and sepsis (11). Recently, the regulation of *CISH* on mouse adiposity, food intake, and glucose metabolism has been reported (12). Meanwhile, chicken *CISH* has been identified as a key gene involved in the growth hormone receptor (GHR) mutation-induced excessive fat deposition which reduced the carcass quality and immunity of broilers to diseases (13). Although genetic variations in porcine *CISH* associated with piglet diarrhea or growth traits have been identified (14, 15), the characteristic of the porcine *CISH* promoter and its upstream transcriptional factors are still unknown.

Integration of RNA-seq and ChIP-seq data in murine erythroid cell lines suggests the existence of signal transducer and activator of transcription 5 (STAT5) and GATA binding protein 1 (GATA1) in CISH promoter (16). The function of GATA1 in intestine immunity or individual growth is limited, while STAT5 is critical in mediating cytokine-driven signaling and exerts widespread effects on immunity, disease, and growth (17–19). For example, the infection of porcine epidemic diarrhea virus (PEDV) upregulates the expression of *STAT3* and *STAT5A* in the porcine intestinal cells (IPEC-J2) (20), and genetic variants in the STAT3/STAT5A/STAT5B region are associated with inflammatory bowel disease (IBD), a complex gastrointestinal disease (21–23). Although genetic variations in *STAT5A* have been reported to associate with bovine milk production (24–26), bovine reproductive performance (24, 27, 28), goat and sheep milk performance (29, 30), and porcine *STAT5A* genetics variations are still poorly understood.

In general, non-synonymous single nucleotide polymorphism (nsSNP), which leads to single amino acid substitution in the coded protein, is of particular interest in mammalian genetic research. For example, nsSNP rs342747498 (T/G) within porcine 17β-hydroxysteroid dehydrogenase type 14 (*HSD17B14)* causes substitution from phenylalanine (Phe) to valine (Val), leading to the alteration of HSD17B14 function on the conversion of gilt estrogen and ovary granular cells apoptosis (31). Meanwhile, using the integrated *in silico* approach, nsSNPs associated with animal economic traits including bovine mastitis resistance (32), chicken abdominal fat content (33), and alpacas classic gray phenotype (34) have been identified. Besides the nsSNP, SNP in the non-coding region of the gene body is equally important, which affects the phenotype by altering the functional gene expression. For example, rs55618224 (T>C) in the 3'UTR of the cell division cycle 42 (CDC42) causes a miR-18a binding-site polymorphism, which impacts the expression of CDC42 in porcine placentas (35). g.32828A>G in the intron of retinoblastoma 1 (RB1) alters gene expression which in turn influences chicken abdominal fat deposition (36).

Given the vital role of CISH in disease and growth, the purpose of the current study was to (1) identify the transcriptional factors of porcine *CISH*, and (2) explore the functional mutations in the identified transcription factor. Herein, we characterized the core promoter of porcine *CISH*, confirmed STAT5A as a trans-activating factor of porcine *CISH*, screened genetic variations in *STAT5A*, and prospected their functions, in attempting to provide novel markers for molecular breeding.

## 2. Materials and methods

### 2.1. Animals and ethics statement

The animals used for genetic variation identification were provided by the Lanxi breeding farm (Lanxi, Heilongjiang, China). A total of 226 Min and 186 Landrace pigs' genomic DNA were used for the SNPs genotyping and association analysis and the phenotype traits of all these animals have been described previously (14). In brief, the piglets' feces were observed and assigned a daily diarrhea score according to the following standard: normal solid feces 0, slight diarrhea with soft and loose feces 1, moderate diarrhea with semi-liquid feces 2, and severe diarrhea with liquid and unformed feces 3 (37). The piglet diarrhea score was calculated as the summation of the daily diarrhea score during the experiment. Additionally, the body weight at birth, 21-day, 28-day, and 35 days were recorded and the average daily gain (ADG) was calculated as follows, ADG = (weight at 35-day - weight at birth)/35.

Animal experimentation was conducted in line with the guidelines for the care and use of animals approved by the Laboratory Animal Management Committee of Northeast Agricultural University (Harbin, Heilongjiang, China).

### 2.2. *In silico* analysis

The core promoters of the porcine *CISH* gene were analyzed using the online neural network promoter prediction (BDGP) software (http://www.fruitfly.org/seqtools/promoter.html). JASPAR software (http://jaspar.binf.ku.dk/cgi-bin/jaspar_db.pl) and PROMO (http://www.urogene.org/cgi-bin/methprimer/methprimer.cgi) were used to search the putative transcription factor binding sites. Functional SNPs in the coding sequence (CDS) of porcine *STAT5A* were predicted using the *in silico* method. The information on nsSNPs in the porcine *STAT5A* gene was first collected from the Ensembl SNP database (http://www.ensembl.org/). Five bioinformatics tools including PhD-SNP (https://snps.biofold.org/phd-snp/), SIFT (http://sift.jcvi.org/), SNAP (http://www.rostlab.org/services/SNAP), Meta-SNP (http://snps.biofold.org/meta-snp/), and PolyPhen-2.0 (http://genetics.bwh.harvard.edu/pph2/uses), which were described in more detail by Emadi et al. (38) and Li et al. (33), were then employed to predict the most deleterious nsSNPs based on incorporating the scores of all the servers. SNPs in the 3'UTR of porcine *STAT5A* were analyzed by miRBase (http://www.mirbase.org) to screen the putative functional SNPs which might alter the microRNA (miRNA) response elements. Similarly, SNPs in the 5' UTR and intron 1 were analyzed by JASPAR and PROMO with a threshold score of 80.

### 2.3. Luciferase reporter constructs and site-directed mutagenesis

To obtain the porcine *CISH* promoter, the 2.5 kb upstream genomic sequence of porcine *CISH* was retrieved from Ensembl, and primer pair CISH-C was designed using Primer 5 software (Supplementary Table S1). The PCR reaction contained 100 ng DNA templates, 0.5 μM of each primer as in Supplementary Table S1, and 10 μL 2 × Taq Master Mix (TaKaRa, Dalian, China). The PCR conditions were 4 min at 94 °C; 35 cycles of 30 s at 94 °C, 30 s at the annealing temperature (Supplementary Table S1), 2 min at 72 °C and a final step at 72 °C for 8 min. PCR products were purified, sequenced, and ligated into PMD18-T (TaKaRa, Dalian, China) to produce PMD18-T-CISH. Then, using PMD18-T-CISH as a template, a series of 5' deletion fragments of *CISH* promoter were amplified using forward primers CISH-C (C1-C5), CISH-P (P1-P3), and the reverse primer CISH-R (Supplementary Table S1). To explore the effect of GATA1 on *CISH* transcription, primer CISH-G was designed and a specific fragment containing putative GATA1 binding element was produced by PCR (Supplementary Table S1). All these PCR products were purified, digested with *Xho* I and *Kpn* I or *Hind* III (TaKaRa, Dalian, China), and inserted into the pGL3-Basic vector (Promega, Madison, Wisconsin, USA) to produce luciferase reporter plasmids pGL3-CISH-C (C1-C5), pGL3-CISH-P (P1-P3) and pGL3-CISH-G.

Using pGL3-CISH-P1 plasmid as a template and the mutagenic primers CISH-Mut1 and CISH-Mut2 (Supplementary Table S1), three mutants of predicted STATx binding sites were generated by PCR. Then these PCR products were purified, digested by restriction endonucleases, and inserted between *Xho* I and *Kpn* I sites of the pGL3-basic vector. All the reconstructed plasmids were confirmed by DNA sequencing and named pGL3-CISH-mut1, pGL3-CISH-mut2, and pGL3-CISH-mut3.

To construct pGL3-STAT5A-Luc and pGL3-STAT5A-SNP1, specific fragments containing different variations in intron 1 of porcine *STAT5A* were amplified with the primer pair STAT5A-Luc (Supplementary Table S1) and the genotyped genomic DNA, inserted into the pGL3-basic vector. Then, using pGL3-STAT5A- Luc plasmid as the template and the mutagenic primers STAT5A-Mut (Supplementary Table S1), the third fragment was produced and the corresponding pGL3-STAT5A-SNP2 was constructed as described above. All the positive plasmids were confirmed by endonuclease digestion and DNA sequencing.

### 2.4. Cell culture, transfection, and luciferase report assay

Hela and IPEC-J2 were selected as *in vitro* model systems to explore porcine CISH core promoters. The cells were cultured in 24-well plates with DMEM supplemented with 10% FBS (Gibco, Carlsbad, CA, USA), maintained at 37 °C in the atmosphere of 5% CO_2_ for 18–24 h. Then, the cells were transfected with 0.5 μg of specific promoter-luciferase plasmid, 0.005 μg of pRL-TK used as an internal control (Promega, Madison, Wisconsin, USA), and 1.5 μL of X-treme GENE HP DNA Transfection Reagent (Roche, USA). Simultaneously, the plasmid pGL3-Basic mixed with pRL-TK was transfected into the corresponding cells as the negative control of the luciferase report assay. After 24–48 h, all the cells were harvested and lysed with the luciferase assay buffer (Promega, Madison, Wisconsin, USA), and the enzymatic activity of firefly and renilla was examined according to Dual-Luciferase Reporter Assay System (Promega, Madison, Wisconsin, USA). Transfection was performed in triplicate and repeated two or three times, and the luciferase activity was calculated as the ratio of firefly to renilla.

### 2.5. Overexpression of porcine *STAT3, STAT5A*, and *GATA1*

The complete coding region of porcine *STAT3* (NM_001044580.1), *STAT5A* (NM_214290.1), and *GATA1* (NM_001278767.1) were amplified using porcine cDNA and primer pairs (Supplementary Table S1) respectively. The purified PCR products were inserted between the *Xho* I and *EcoR* I or *Not* I sites of the pCMV-HA vector (Promega, Madison, WI, USA) to construct pCMV-HA-STAT3, pCMV-HA-STAT5A, and pCMV-HA-GATA1, respectively. These expression plasmids were confirmed by DNA sequencing and Western blot analysis. Then, IPEC-J2 cells were transfected with 0.25 μg of *CISH* promoter luciferase reporter plasmid (pGL3-CISH-C4, pGL3-CISH-P1, or pGL3-CISH-G), 0.25 μg of corresponding expression plasmid (pCMV-HA-STAT3, pCMV-HA-STAT5A, pCMV-HA-GATA1, or pCMV-HA), and 0.005 μg of pRL-TK. Relative luciferase activity was calculated as described above.

To validate the role of transcriptional factors on porcine *CISH* mRNA expression, IPEC-J2 cells were transfected with 2 μg of pCMV-HA-STAT3, pCMV-HA-STAT5A, pCMV-HA-GATA1, or pCMV-HA, respectively. All the cells were collected, and RT-qPCR was performed.

### 2.6. Western blot, RNA extraction, and RT-qPCR

For the Western blot, IPEC-J2 cells transfected with pCMV-HA-STAT3, pCMV-HA-STAT5A, pCMV-HA-GATA1, or pCMV-HA were lysed with RIPA buffer (SEVEN, Beijing, China), boiled with 5 × denaturing loading buffer, resolved in 12% SDS-PAGE, and transferred to Immuno-Blot polyvinylidene fluoride (PVDF) membrane (Millipore, Billerica, MA, USA). Then, the membrane was blocked with 5% BSA, washed with TBST, and incubated with HA-tag antibody (ABclonal, Wuhan, China) in 1:1000 dilution for 12 h at 4 °C, and a secondary antibody at room temperature for 2 h. Lastly, this incubated membrane was washed with TBST and Super ECL kit (SEVEN, Beijing, China) was used to show the bands.

For the RNA extraction and RT-qPCR, total RNAs were extracted from IPEC-J2 cells using TRIzol (Takara, Dalian, China) and reverse transcribed into cDNA using PrimerScript RT Master Mix (Takara, Dalian, China). According to SYBR Premix Ex Taq (Takara, Dalian, China), RT-qPCR was conducted on an ABI 7500 system (Applied Biosystems, Foster City, CA, USA) with the following reaction: 100 ng cDNA, 0.2 μM of each primer (Supplementary Table S1), and 10 μL SYBR mix (Takara, Dalian, China). The qPCR conditions were 95 °C for 30 s, 40 cycles with 95 °C for 5 s, and 60 °C for 35 s, with the melting curves constructed at the same time. In line with the 2^−Δ*ΔCT*^ method (35), Glyceraldehyde-3-phosphate dehydrogenase (GAPDH) (36, 37) was selected as a housekeeping gene to normalize the expression of target genes and relative mRNA expression of a specific gene in cells was calculated.

### 2.7. Identification and genotyping of variations in porcine *STAT5A*

The 5'UTR and 3'UTR of porcine *STAT5A* were amplified using the primer pair STAT5A-5'UTR or STAT5A-3'UTR (Supplementary Table S1) and the mixed genomic DNA pools which consisted of five healthy Min, five diarrheal Min, five healthy Landrace, and five diarrheal Landrace pigs described above. All the PCR products were purified, sequenced commercially (Sangon, Shanghai, China), and aligned with Clustal Omega. The predicted functional SNPs by *in silico* tools were genotyped in Min and Landrace populations through PCR-based single-strand conformation polymorphism (PCR-SSCP). In brief, fragments containing SNPs were amplified by PCR using primer pair STAT5A-SNP (Supplementary Table S1). Then 1 μL PCR products mixed with 9 μL denaturation buffer were denatured for 10 min at 98 °C, placed on iced water for 5 min, separated on a 14% PAGE gel, and resolved by silver staining. PCR products with diverse genotypes were purified and sequenced commercially (Sangon, Shanghai, China).

### 2.8. Statistical analysis

The significant difference was evaluated using the *t-*test in GraphPad Prism 5 software (GraphPad, La Jolla, CA, USA) or one-way ANOVA. The observed heterozygosity (*Ho*), expected heterozygosity (*He*), effective allele numbers (*Ne*), and chi-square test for Hardy-Weinberg equilibrium of the genetic variation polymorphisms were calculated using Popgene software (version 1.32). The Haploview 4.2 software was used to analyze the linkage disequilibrium (LD) between g.508A>C and g.566C>T and the haplotype was constructed.

A mixed linear model procedure of SAS version 8.0 was used to perform association analysis between SNP or haplotype and the phenotype trait in Min pigs. The following statistical model was adopted:


$$\begin{array}{l} Y_{ij}\;=\mu +G_{\text{i}}+S_{j}+\;e_{ij} \end{array}$$

Where *Y*_*ij*_ is the observed phenotype traits, μ is the population mean, *G*_*i*_ is the fixed effect of genetics, *S*_*j*_ is the random effect of the sow, and *e*_*ij*_ is the random residual.

## 3. Results

### 3.1. Identification of core promoter of porcine *CISH*

**Figure 1:**
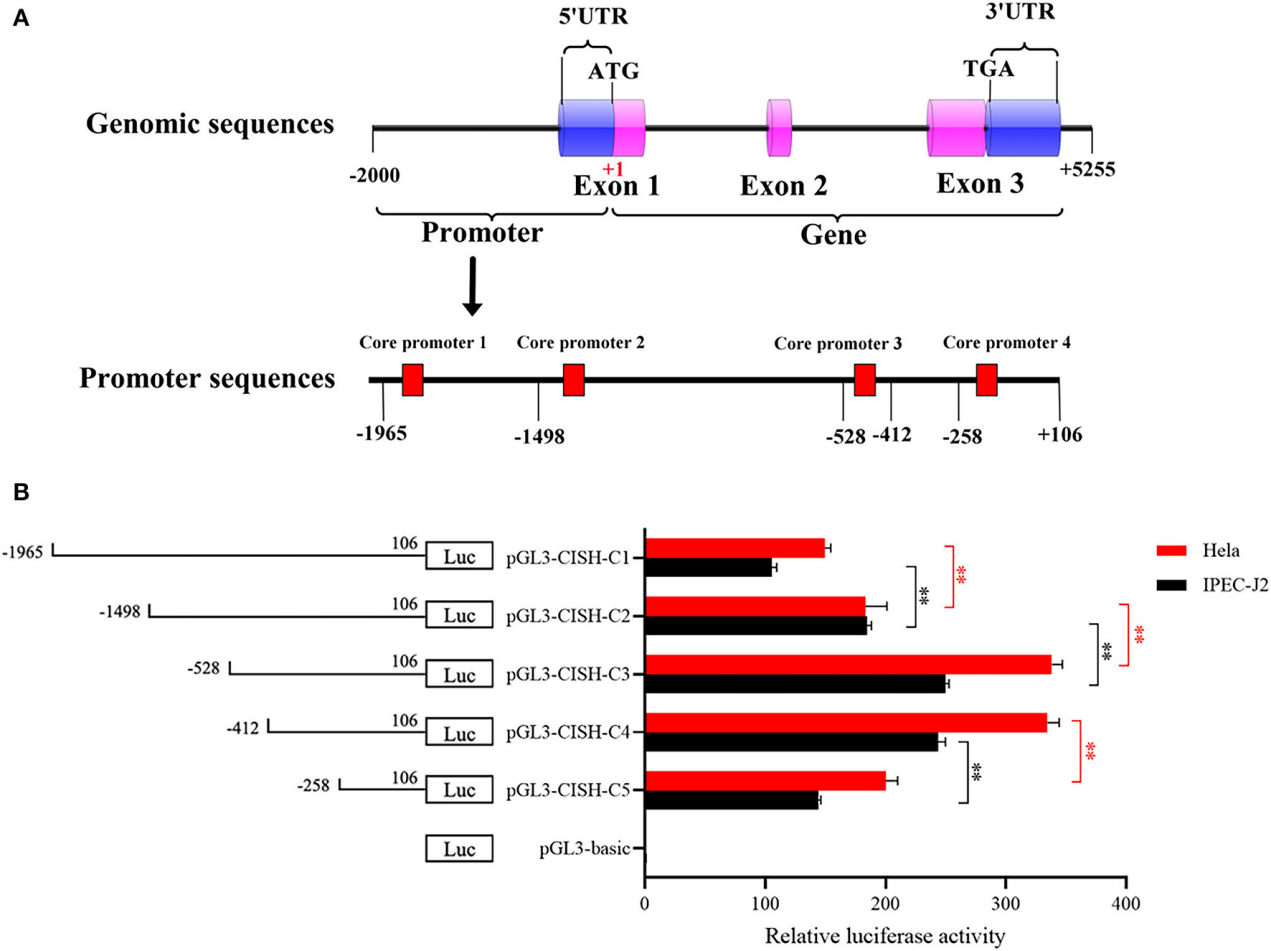
5'-Deletion analysis of the CISH promoter. **(A)** Schematic diagram of the predicted core promoter regions in the CISH promoter. **(B)** Promoter activities of a series of deleted constructs determined by luciferase assay. Left panel, schematic representation of the 5'-deletions linked with luciferase gene in pGL3 vector. The nucleotides are numbered from the potential translational start site (ATG) that was assigned +1. Right panel, the relative activities of five 5' deleted constructs to the pGL3-basic determined by luciferase assays. Relative luciferase activity was represented by normalizing to pRL-TK and then normalized against the activity of pGL3-basic in IPEC-J2 or Hela cells. Values are shown as means ± SE of three replicates. Statistical differences in relative activities were analyzed in the same cells. **P < 0.01.

A 2071-bp fragment containing a 5'-flanking region and 106-bp coding sequence was produced by PCR, and four putative core promoters in the region of −1,807/−1,758, −1,358/−1,309, −460/−411, and −201/−152 were predicted by BDGP online software (Figure 1A). Subsequently, five 5'-deletions were amplified and inserted into the pGL3 plasmid to produce the *CISH* promoter luciferase reporter gene plasmids. As shown in Figure 1B, all these plasmids had luciferase activity in both IPEC-J2 and Hela cells. Further analysis showed the CISH-C4 fragment (sequence from −412 to 106 bp) had higher activity than the CISH-C5 fragment (sequence from −258 to 106 bp), suggesting the existence of important positive regulatory transcription elements in −412/−258 bp. However, the activity of porcine CISH-C2 (sequence from −1,498 to 106 bp) was lower than CISH-C3 (from −528 to 106 bp), indicating there were elements in the region of −1,498/−528 that could negatively regulate the transcription of porcine *CISH*.

### 3.2. Identification of regulatory elements of porcine *CISH*

**Figure 2:**
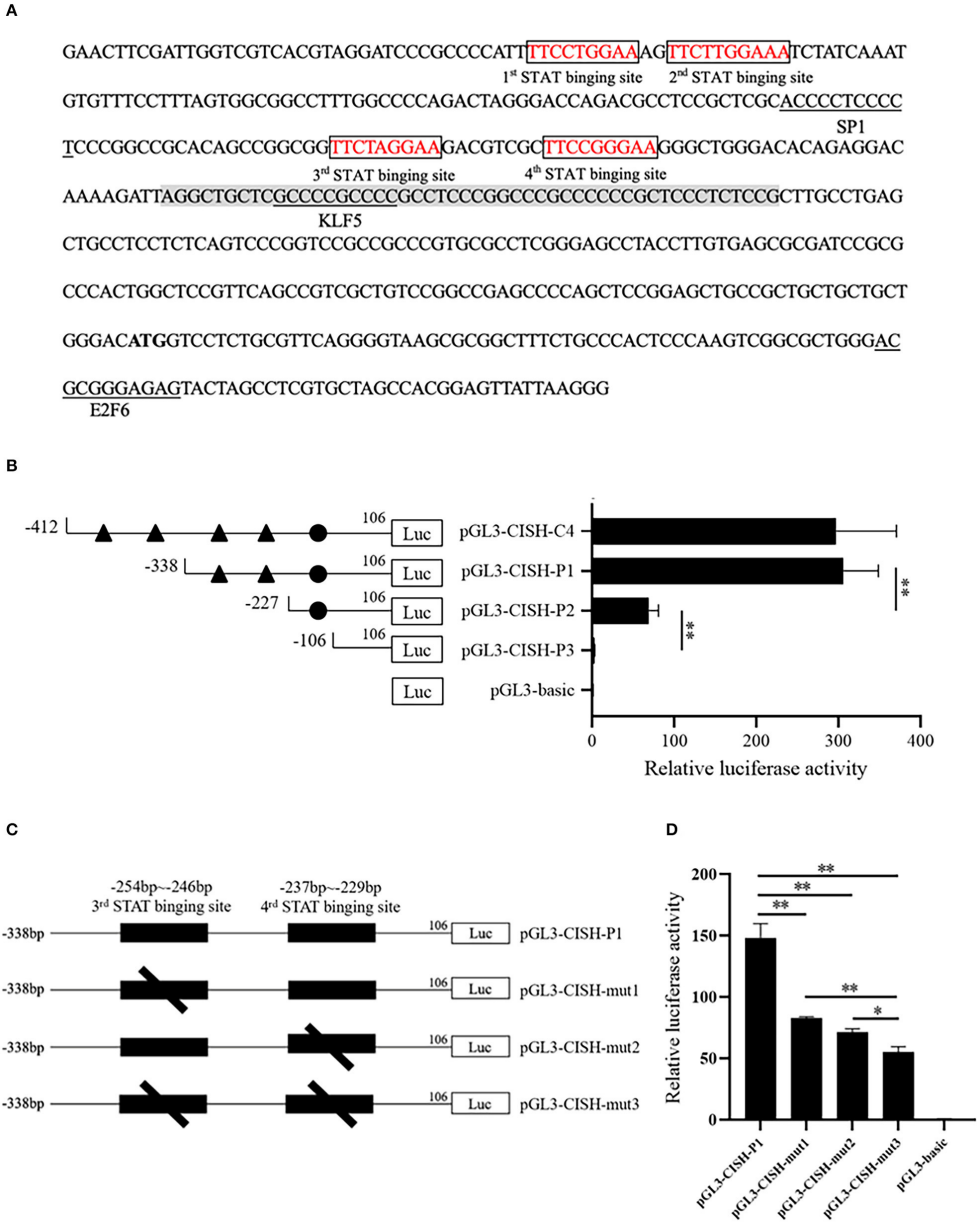
STATx binding sites in the CISH promoter. **(A)** The predicted transcriptional factors and core promoter in the CISH-C4 fragment. **(B)** 5'-Deletion analysis of the CISH-C4 fragment. **(C)** Schematic presentation of two putative STAT-binding sites and deletion mutants for each of the sites. **(D)** Site-directed mutagenesis in the STATx binding sites by luciferase assay. Results are expressed as ratios of relative activities that were represented by normalizing to pRL-TK and then normalized against the activity of pGL3-basic in IPEC-J2 cells. Values are shown as means ± SE of three replicates and the statistical differences were analyzed in the same cells. *P < 0.05, **P < 0.01.

*In silico* analysis showed the CISH-C4 fragment contained one core promoter and tetramer STATx transcription factor binding sites (Figure 2A). To verify this prediction, three 5'-deletion plasmids were constructed. Luciferase activity analysis showed the deletion of the first two putative STATx binding sites (−412/−338) did not alter the luciferase activity of fragment CISH-C4 (Figure 2B). However, the deletion of the third and fourth STATx binding sites (−338/−227) significantly reduced the luciferase activity of CISH-C4 (Figure 2B). Additionally, as shown in Figure 2B, the disappeared luciferase activity of the CISH-P3 fragment indicated the existence of a minimal core promoter in the region of −227/−106. To verify the existence of STATx binding sites, the predicted third or fourth STATx binding sites within the CISH-P1 fragment were mutated (Figure 2C). Luciferase activity analysis revealed that the mutations reduced the luciferase activity of CISH-P1 significantly (Figure 2D).

### 3.3. Effect of STAT3 and STAT5A on porcine *CISH* transcriptional activity and mRNA expression

**Figure 3:**
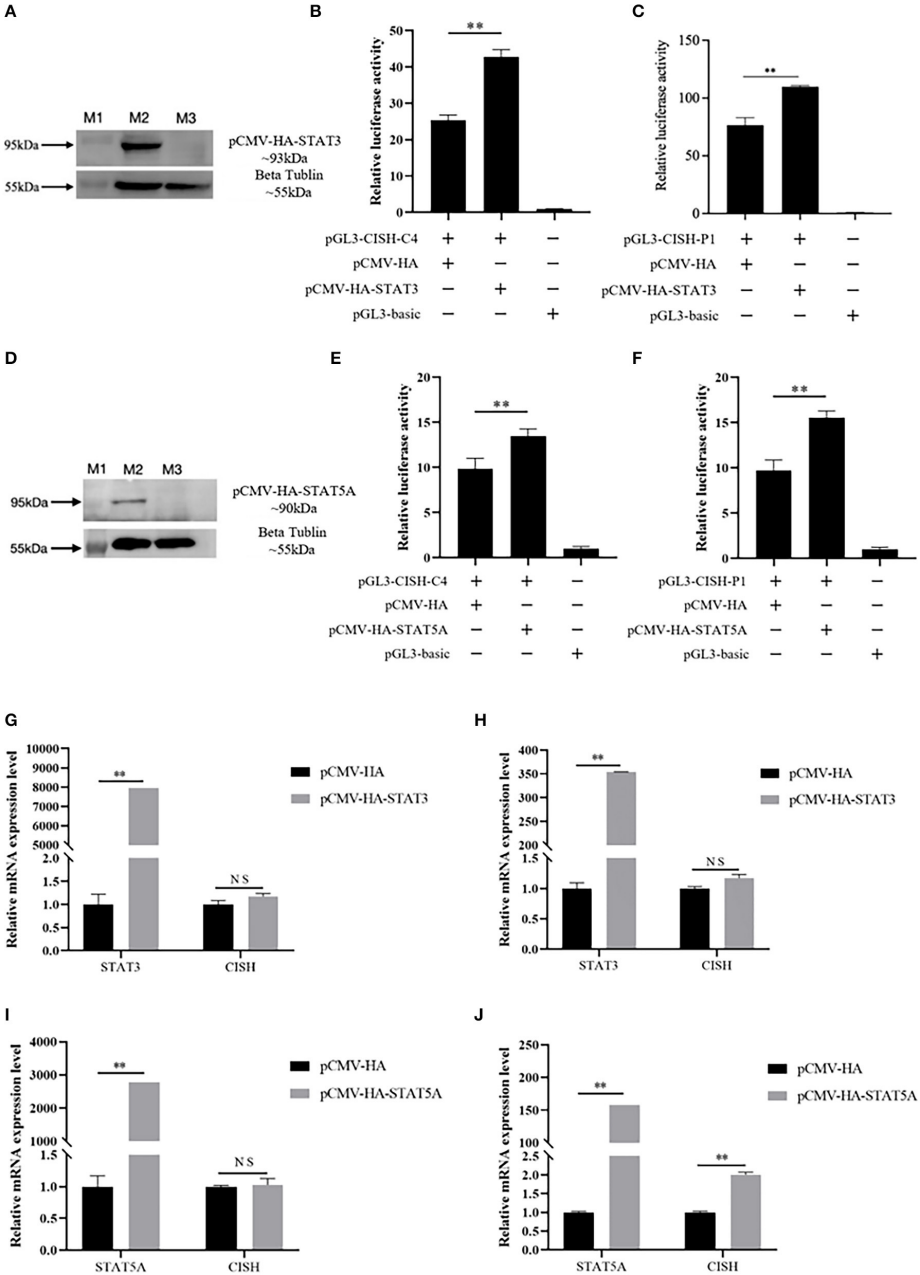
Effect of porcine STAT3 and STAT5A on CISH transcriptional activity and mRNA expression. **(A)** Over expression of porcine STAT3 in IPEC-J2. Lane M1, protein marker. Lane M2, the expressed protein of pCMV-HA-STAT3; Lane M3, control of pCMV-HA. **(B)** Overexpression of STAT3 up-regulates CISH-C4 activity in IPEC-J2. **(C)** Overexpression of STAT3 up-regulates CISH-P1 activity in IPEC-J2. **(D)** Overexpression of porcine STAT5A in IPEC-J2. Lane M1, protein marker. Lane M2, the expressed protein of pCMV-HA-STAT5A; Lane M3, control of pCMV-HA. **(E)** Overexpression of STAT5A up-regulates CISH-C4 activity in IPEC-J2. **(F)** Overexpression of STAT5A up-regulates CISH-P1 activity in IPEC-J2. **(G, H)** Overexpression of STAT3 has no effect on CISH mRNA expression in IPEC-J2. **(I)** Overexpression of STAT5A has no effect on CISH mRNA expression after 24 h in IPEC-J2. **(J)** Overexpression of STAT5A increased CISH mRNA expression after 48 h in IPEC-J2. Values are shown as the mean ± SD (*n* = 3). **P < 0.01.

In this study, based on the literature, porcine STAT3 and STAT5A were selected to verify their effect on *CISH* transcriptional activity and mRNA expression. The expression plasmid pCMV-HA-STAT3 or pCMV-HA-STAT5A, together with *CISH* promoter luciferase reporter plasmids pGL3-CISH-C4 or pGL3-CISH-P1, were transfected into IPEC-J2. The luciferase activity analysis showed that the overexpression of STAT3 could significantly increase *CISH* transcriptional activity (*P* < 0.01) (Figures 3A–C). Similarly, the overexpression of STAT5A up-regulated *CISH* transcriptional activity (*P* < 0.01) (Figures 3D–F). The RT-qPCR revealed that the overexpression of STAT3 did not affect *CISH* mRNA expression (Figures 3G, H). However, STAT5A promoted the *CISH* expression at 48 h after transfection (*P* < 0.01) (Figures 3I, J).

### 3.4. Effect of GATA1 on porcine *CISH* transcriptional activity and mRNA expression

**Figure 4:**
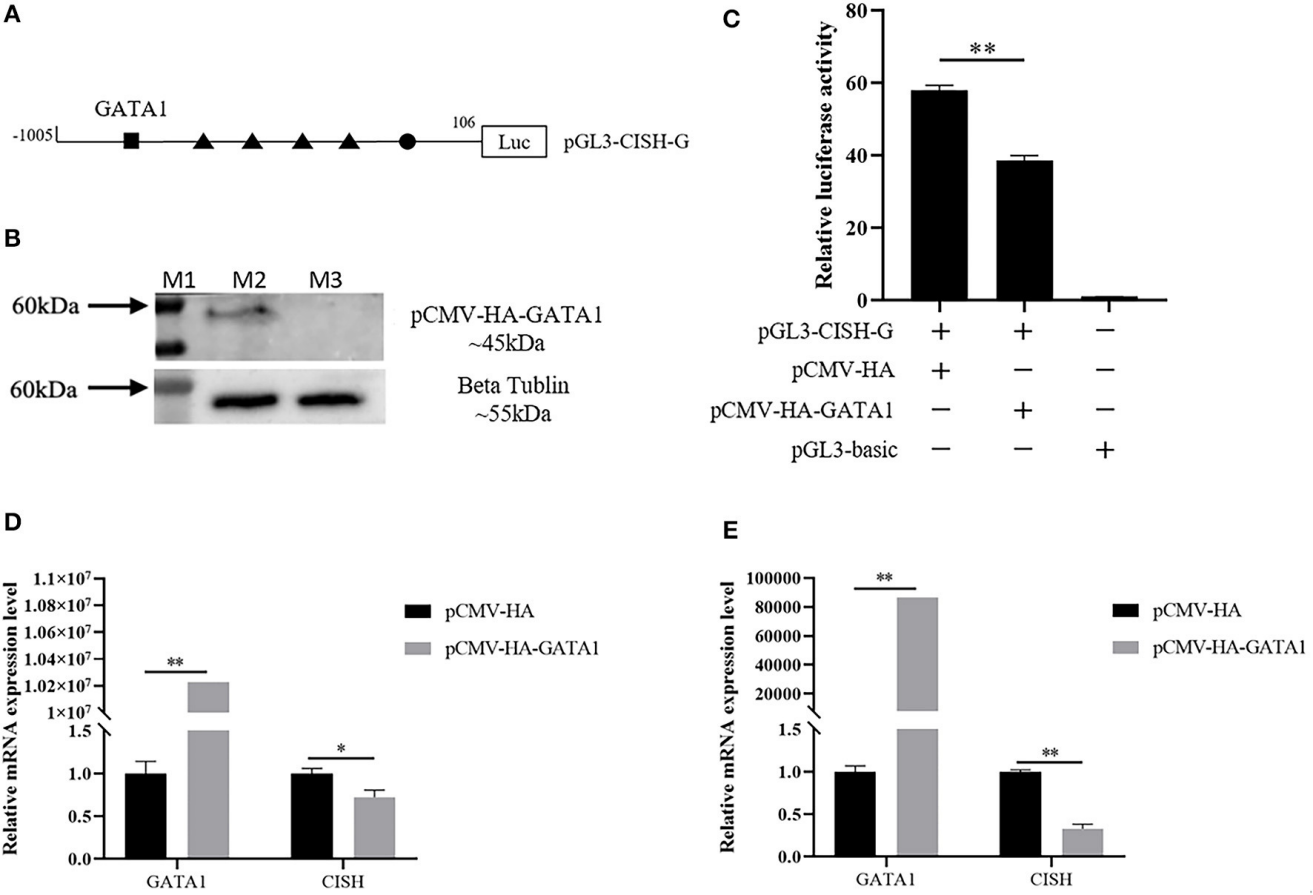
Effect of porcine GATA1 on CISH transcriptional activity and mRNA expression. **(A)** GATA1 binding sites in the CISH promoter. **(B)** Over expression of porcine GATA1 in IPEC-J2. **(C)** Overexpression of GATA1 down-regulates CISH-G activity in IPEC-J2. **(D)** Overexpression of GATA1 down-regulates CISH mRNA expression in IPEC-J2 after transfection for 24 h. **(E)** Overexpression of GATA1 down-regulates CISH mRNA expression in IPEC-J2 after transfection for 48 h. Values are shown as the mean ± SD (*n* = 3). *P < 0.05, **P < 0.01.

*In silico* analysis showed the CISH-G fragment containing GATA1 binding sites upstream of the tetramer STATx transcription factor binding sites (Figure 4A). The reconstruction plasmid pCMV-HA-GATA1 and pGL3-CISH-G were transfected into IPEC-J2. The luciferase activity analysis showed that GATA1 overexpression significantly repressed *CISH* transcriptional activity (*P* < 0.01) (Figures 4B, C). The RT-qPCR revealed that the overexpression of GATA1 reduced the *CISH* mRNA expression at 24 and 48 h after transfection (Figures 4D, E).

### 3.5. Identification of the putative function SNPs in porcine *STAT5A*

In this study, a total of 49 SNPs in the porcine *STAT5A* coding region were retrieved from the Ensembl database. Among the 14 nsSNPs, only c.870G>T was the predicted deleterious nsSNP with Polyphen-2 (Supplementary Table S2). Then, a 1618-bp fragment containing a 210-bp intron17, 1384-bp exon18 (containing 1221-bp 3'UTR), and 24-bp downstream region of porcine *STAT5A* was amplified. Although 9 SNPs were identified in this fragment, none of these SNPs was predicted to alter the miRNA binding sites (data not given). Similarly, a 909-bp fragment containing 231-bp 5'UTR and 678-bp intron1 of porcine *STAT5A* was amplified and three SNPs (g.373C>G, g.508A>C, and g.566C>T) were identified in intron 1. According to JASPAR, g.373C>G caused a zinc finger protein 454 (ZNF454) polymorphism; g.508A>C was predicted to alter the binding site of E2F transcription factor 4 (E2F4) or transcription factor Dp-1 (TFDP1), where E2F4 or TFDP1would bind to the C allele but not the A allele; g.566C>T altered the binding sites of PR domain containing 4 (PRDM4) or RELB proto-oncogene, and NF-kB subunit (RELB) (Supplementary Table S3). However, among these SNPs, PROMO revealed only g.566C>T altered the binding of ETS transcription factor ELK1 (Elk-1) (Supplementary Table S2). Collectively, the *in silico* analysis indicated that g.508A>C or g.566C>T were the putative functional SNPs.

### 3.6. Genotyping and association analysis in Min and Landrace population

**Figure 5:**
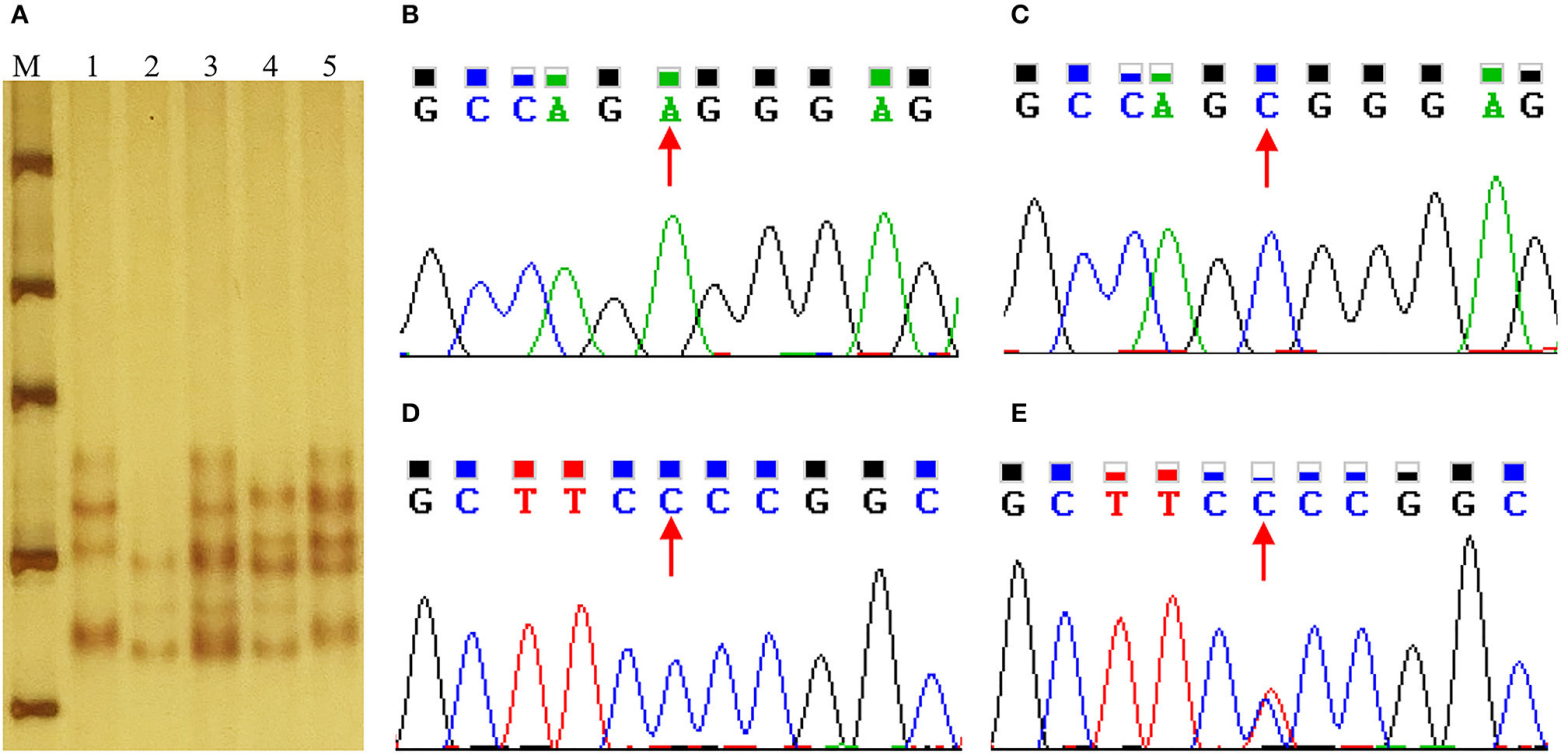
Sequence and genotyping of g.508A>C and g.566C>T in the porcine STAT5A promoter region. **(A)** Genotyping of g.508A>C and g.566C>T in the intron 1 of porcine STAT5A. Lane M molecular marker DL1000; Lanes 1~5 show the genotype of AACC, CCCC, ACCC, CCTC and ACTC. **(B, C)** Sequence of g.508A>C in porcine STAT5A intron 1. **(D, E)** Sequence of g.566C>T in intron 1 of porcine STAT5A.

g.508A>C and g.566C>T were genotyped by PCR-SSCP in Min and Landrace populations. As shown in Figure 4, three genotypes (AA, AC, and CC) of g.508A>C (Figures 5A) were observed, while only CC and CT genotypes of g.566C>T (Figures 5A, D, E) were found. The frequency of the A allele (g.508A>C) was 0.61 in Min pigs, and the C allele (g.566C>T) was 0.94 in this population. However, these two alleles were fixed in the Landrace population. In Min pigs, the *Ho* and *He* of g.508A>C were 0.49 and 0.48, while *Ho* and *He* of g.566C>T were 0.11 and 0.11, respectively; the effective allele number (*Ne*) of g.508A>C and g.566C>T was 1.91 and 1.12, respectively; and the polymorphism information content (*PIC*) values were 0.36 and 0.10, respectively (Table 1). Statistical analysis showed g.508A>C was not associated with Min piglets' phenotype traits, while animals with CC genotype at g.566C>T had more 28-day body weight, 35-day body weight, and ADG than those of CT genotype (*P* < 0.05) (Table 2). Additionally, three haplotypes were constructed and Hap1:AC was the major haplotype with a frequency of 0.61, while the minor Hap3:CT was 0.06 (Table 3). Association analysis indicated Min piglets with Hap1:AC had higher 28-day body weight, 35-day body weight, and ADG when compared to Hap3:CT (*P* < 0.05) (Table 3).

**Table 1:** Genotype and allelic frequency of g.508A>C and g.566C>T in Min pig and Landrace populations.

**SNP**	**Breed**	**Genotype** **frequency**	**Allelic frequency**	**H_0_**	**He**	**Ne**	**PIC**	***χ^2^*(HWE)**
g.508 A>C rs345257049	Min	AA 0.36 (80) AC 0.49 (108) CC 0.15 (32)	A 0.61 C 0.39	0.49	0.48	1.91	0.36	0.21
	Landrace	AA 1 (180) AC 0 (0) CC 0 (0)	A 1 C 0	0	0	1	0	0
g.566 C>T rs333118906	Min	CC 0.89 (195) TC 0.11 (25) TT 0 (0)	C 0.94 T 0.06	0.11	0.11	1.12	0.10	0.80
	Landrace	CC 1 (180) TC 0 TT 0	C 1 T 0	0	0	1	0	0

H_0_ observed heterozygosity, He expected heterozygosity, Ne effective allele numbers, PIC polymorphism information content, χ^2^(HWE) chi-square test for the Hardy–Weinberg equilibrium.

**Table 2:** Association between g.508A>C or g.566C>T with phenotype in Min pig population.

**SNPs**	**Traits**	**Genotypes**		
		**AA**	**AC**	**CC**
g.508 A>C rs345257049	Number	80	108	32
	Diarrhea score	2.96 ± 0.29	2.53 ± 0.24	2.81 ± 0.45
	Birth weight (kg)	1.03 ± 0.03	1.03 ± 0.02	1.12 ± 0.04
	21-day body weight (kg)	3.48 ± 0.11	3.23 ± 0.09	3.54 ± 0.17
	28-day body weight (kg)	4.59 ± 0.14	4.27 ± 0.12	4.43 ± 0.22
	35-day body weight (kg)	5.81 ± 0.17	5.41 ± 0.14	5.63 ± 0.27
	ADG (kg•d^−1^)	0.14 ± 0.00	0.12 ± 0.00	0.13 ± 0.01
		**CC**	**TC**	**TT**
g.566 C>T rs333118906	Number	195	25	0
	Diarrhea score	2.74 ± 0.18	2.63 ± 0.50	-
	Birth weight (kg)	1.05 ± 0.02	0.99 ± 0.04	-
	21-day body weight (kg)	3.40 ± 0.07	3.05 ± 0.19	-
	28-day body weight (kg)	4.47 ± 0.09^a^	3.95 ± 0.24^b^	-
	35-day body weight (kg)	5.66 ± 0.11^a^	4.98 ± 0.29^b^	-
	ADG (kg•d^−1^)	0.13 ± 0.00^a^	0.11 ± 0.01^b^	-

Different lowercase letters indicated significant difference (*P* < 0.05) and capital letters indicated significant difference (*P* < 0.01).

**Table 3:** Association between haplotypes with phenotype in Min pig population.

**Traits**	**Hap1: AC**	**Hap2: CC**	**Hap3: CT**
Number	268	147	25
frequency	0.61	0.33	0.06
Diarrhea score	2.78 ± 0.16	2.64 ± 0.21	2.62 ± 0.50
Birth weight (kg)	1.03 ± 0.01	1.08 ± 0.02	0.99 ± 0.04
21-day body weight (kg)	3.37 ± 0.06	3.39 ± 0.08	3.05 ± 0.19
28-day body weight (kg)	4.46 ± 0.07^a^	4.40 ± 0.10	3.95 ± 0.24^b^
35-day body weight (kg)	5.64 ± 0.09^a^	5.59 ± 0.12	4.98 ± 0.29^b^
ADG (kg•d^−1^)	0.13 ± 0.00^a^	0.13 ± 0.00	0.11 ± 0.01^b^

Different lowercase letters indicated significant difference (*P* < 0.05).

### 3.7. Effect of SNPs on porcine *STAT5A* transcriptional activity

**Figure 6:**
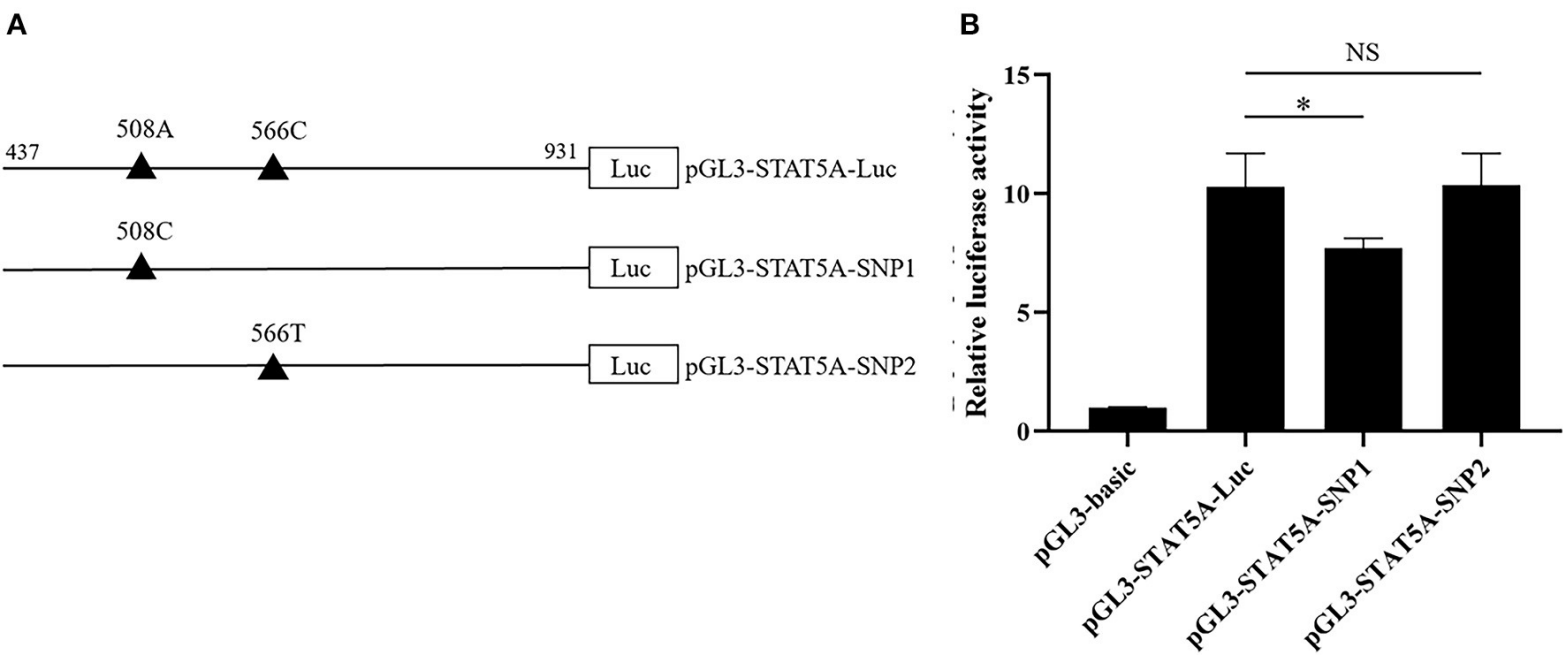
The effects of g.508A>C or g.566C>T on the porcine STAT5A transcriptional activity. **(A)** Schematic presentation of the luciferase reporter construct. SNPs were named and numbered from the first nucleotide of the first exon of porcine STAT5A (ENSSSCT00000064828.3), which was assumed as the putative transcriptional initial site and assigned as +1. **(B)** Luciferase assays of different SNPs luciferase reporter constructs in IPEC-J2 cells. Values are shown as the mean ± SD (*n* = 3). *P < 0.05.

As shown in Figure 6A, the STAT5A promoter luciferase reporter pGL3-STAT5A-Luc (g.508A and g.566C), pGL3-STAT5A-SNP1 (g.508C and g. 566C), and pGL3-STAT5A-SNP2 (g.508A and g.566T) were constructed to verify the putative function of g.508A>C or g.566C>T. After transfection, the luciferase activity of these reporter vectors was calculated and compared. The statistical analysis indicated the luciferase activity of g.508A>C A allele was higher than that of the C allele (*P* < 0.05), while the difference between the C and T allele of g.566C>T was not significant (Figure 6B).

## 4. Discussion

Porcine *CISH* was widely expressed in multiple tissues including the small intestine of piglets and adult pigs (15, 39), which indicated *CISH* functioned in pig intestinal physiology. In this study, Hela and IPEC-J2 were selected as *in vitro* models to identify the *CISH* core promoter. Hela is a general cell model widely used in biology research. IPEC-J2, a jejunum epithelium cell line isolated from a neonatal piglet, has been used for studying porcine intestinal healthy (40). Interestingly, the highest luciferase activity of the CISH −412/−258 region was presented in these two cell lines, suggesting the existence of positive regulatory transcription elements. The following bioinformatics, 5'-deletion analysis, and site-directed mutagenesis verified the existence of STATx binding sites in porcine *CISH* promoter, which was consistent with the study in mouse *CISH* promoter where two sets of conserved tandem STAT5 binding sites were identified (41, 42).

Among the seven numbers of the STAT family, *STAT3* and *STAT5*, related to intestinal mucosal immune responses, are included in the candidate loci linked to IBD susceptibility (21–23). *STAT5* is comprised of *STAT5A* and *STAT5B* which are organized along chromosomes from head to head. Although, *STAT5A* and *STAT5B* showed sequence similarities of more than 90%, targeted distinctive phenotypes, or functions (43, 44). A recent study on host response to PEDV infection in IPEC-J2 revealed the high expression of the STAT family except *STAT5B* and *STAT6* after infection (20). Considering the role of *CISH* in immunity and disease, STAT3 and STAT5A were selected to estimate their effects on porcine CISH transcription and expression in this study. We found these two factors positively regulated the transcription of *CISH* in IPEC-J2, while the increased *CISH* mRNA expression was found only in STAT5A overexpressed cells. Interestingly, the overexpression of GATA1 down-regulated the transcription and expression of *CISH* in IPEC-J2 at 24h after transfection, while STAT5A up-regulated *CISH* at 48 h rather than 24 h. Existing studies proved that STAT5 was involved in signal transduction from multiple factors, and the phosphorylation of STAT5 resulted in dimerization, translocation to the nucleus, and DNA binding to target binding sites in promoters and enhancers (45, 46). Hence, the potential explanation is STAT5A needs to be phosphorylated before rapidly regulating downstream target genes.

To enhance the accuracy of the *in silico* method, it was recommended to use at least four or five tools to obtain consistent measures of the effects of nsSNPs on the target protein structure and function (38). However, using this strategy, the most deleterious nsSNP within *STAT5A* was identified. The nsSNPs studied in this research were retrieved from the public database with limited information. Sequencing *STAT5A* coding region using genomic DNA with different genetic backgrounds is suggested to provide more SNPs information.

A functional SNP in porcine *STAT3* promoter associated with piglet diarrhea has been identified (47), while none of the genotyped SNPs in *STAT5A* affected piglet diarrhea. Considering the contribution of *STAT3* to the replication of PEDV (48), the current study indicates that *STAT3* might be the candidate gene for piglet diarrhea. For the performance trait, Min pigs with CC genotype in g.566C>T had higher body weight at weaning and ADG. Interestingly, the C allele was fixed in Landrace, and the C allele frequency was 0.94 in Min pigs. The Min is a Chinese local breed with a lower growth rate when compared with Landrace, a general breed selected for performance traits (http://afs.okstate.edu/breeds/swine/minzhu/index.html/). The finding of this study is consistent with the characteristics of these two breeds, and it is suggested to eliminate the T allele to advance the improvement of the Min pig's growth traits.

*In silico* tools revealed both g.508A>C and g.566C>T cause transcriptional polymorphism, however, the subsequent luciferase reporter assay verified that only g.508A>C, which was predicted to alter E2F4 or TFDP1 transcriptional factor binding sites, showed diverse transcriptional activity. According to JASPAR, the C allele of g.508A>C owned an E2F4 or TFDP1 binding site, and the lower luciferase activity of the C allele was found. Whether E2F4 or TFDP1 contribute to *STAT5A* transcription through g.508A>C polymorphism needs to be verified by exploring the interaction between these proteins and the *STAT5A* promoter. For the g.566C>T, although this SNP was associated with phenotype, the consistent luciferase activity of diverse alleles indicated that this SNP might be a linked marker. In the future, more explorations in the *STAT5A* promoter are suggested to understand the mechanism of *STAT5A* transcriptional regulation and provide novel causative molecular markers for selection breeding.

## 5. Conclusion

STAT5A positively regulates porcine *CISH* transcription and expression in the IPEC-J2 cell. *STAT5A* could be selected as a candidate gene for the Min piglet growth trait. The intronic mutation g.566C>T was associated with the Min piglet growth trait.

## Data availability statement

The original contributions presented in the study are included in the article/Supplementary material, further inquiries can be directed to the corresponding author.

## Ethics statement

The animal study was reviewed and approved by Laboratory Animal Management Committee of Northeast Agricultural University.

## Author contributions

BN and DG designed and supervised the experiment. DY, ZC, YZ, XG, and GX conducted the experiment. DY, YZ, and ZC analyzed the data. XW, SD, and JC provided the animals. BN, DG, DY, and XY contributed to the writing of the manuscript. All authors contributed to the article and approved the submitted version.
