# Supplementary material for: Identification of mutations in porcine STAT5A that contributes to the transcription of CISH
Source: Front Vet Sci. 2023 Jan 17;9:1090833. doi: 10.3389/fvets.2022.1090833 (PMC9887310; doi:10.3389/fvets.2022.1090833)
Supplement: Supplementary file 1 [file Data_Sheet_1.docx]

**Table S1.** Primers used for PCR, RT-qPCR, SNPs identification and plasmids construction

| **Prime** | **Primer sequence (5’-3’)** | **Product**  **size(bp)** | **Annealing**  **temp(℃)** | **Binding**  **region** |
| --- | --- | --- | --- | --- |
| CISH-C1 | F:ATAGGTACCGAGCTTGGGATTTGTCTCC | 2071 | 62 | -1965 |
| CISH-C2 | F:TATGGTACCAGTCTGTCATCCCTGCTTG | 1604 | 62 | -1498 |
| CISH-C3 | F:ATCGGTACCTTCAACTCGGTCTTTCCC | 634 | 63 | -528 |
| CISH-C4 | F:AACGGTACCGAACTTCGATTGGTCGTCA | 518 | 63 | -412 |
| CISH-C5 | F:TAAGGTACCGCGGTTCTAGGAAGACGT | 364 | 63 | -258 |
| CISH-R | R:AGACTCGAGCCCTTAATAACTCCGTGGC | - | - | Intron 1 |
| CISH-P1 | F:TATGGTACCTCCTTTAGTGGCGGCCTTTG | 444bp | 61 | -338 |
| CISH-P2 | F:ATAGGTACCGGCTGGGACACAGAGGACAA | 333bp | 61 | -227 |
| CISH-P3 | F:ATAGGTACCCCTCGGGAGCCTACCTTGT | 212bp | 61 | -106 |
| CISH-Mut1 | F:GCACAGCCGGCGGGTTTAGGAAGACGTCGCT | - | - | - |
|  | R:AGCGACGTCTTCCTAAACCCGCCGGCTGTGC | - | - | - |
| CISH-Mut2 | F:AGACGTCGCTTCCGGACTGGGCTGGGACACA | - | - | - |
|  | R:TGTGTCCCAGCCCAGTCCGGAAGCGACGTCT | - | - | - |
| CISH-G | F: ACCCTCGAGGCCTCAGATCGGTAGTCATT | 1111 | 59 | -1005 |
|  | R: CCCAAGCTTCCCTTAATAACTCCGTGGC |  |  | Intron 1 |
| *STAT3* | F:GAATTCCCATGGCCCAATGGAATCAGCTACAG | 2313bp | 58 | - |
|  | R:AAACTCGAGTCACATGGGGGAGGTAGCGCAC |  |  |  |
| *STAT5A* | F:GCTCGAGTTATGGCGGGCTGGATCCAG | 2400bp | 62 | - |
|  | R:TAATAATGCGGCCGCTCAGGAGAGTGAGCCCCT |  |  |  |
| *GATA1* | F:CGGAATTCTTATGGAGTTCCCTGGCCT | 1239bp | 58 | - |
|  | R:AATCTCGAGTCACGAGCTGAGCGGGGC |  |  |  |
| CISH-Exp | F:CCCAGCCCATCCAGAGAGTGAG | 102 | 56 | - |
|  | R:CAATACCAGCCAGATTCCCGAAGG |  |  |  |
| STAT3-Exp | F:ATCTCCAGGATGACTTTGAT | 203 | 56 | - |
|  | R:AGTTTTCTGCACATACTCCA |  |  |  |
| STAT5A-Exp | F:GCAGTCCTGGTGTGAGAAGTTGG | 146 | 57 | - |
|  | R:TGATGTCTGTGATGGTGGCGTTG |  |  |  |
| GATA1-Exp | F:ATTGTCAGCAAACGGGCAGGTAC | 148 | 57 | - |
|  | R:GCATGGTCAGTGGTCGGTTCAC |  |  |  |
| GAPDH-Exp | F:CCCCAACGTGTCGGTTGT | 83 | 55 | - |
|  | R:CCTGCTTCACCACCTTCTTGA |  |  |  |
| STAT5A-3’UTR | F: GCGGTGAGCAATGAGACT | 1618 | 55 | Intron 17 |
|  | R: TGGAGCCATACCAGCAAA |  |  | +22413 |
| STAT5A-5’UTR | F:AGGGCGGAGGCAGGGAGAAA | 909 | 60 | Exon 1 |
|  | R:CTGTGCGGGAAGAAAGATGGTGA |  |  | Intron 1 |
| STAT5A-SNP | F:GAGGGAGGGAGGGGTCGA | 233 | 58 | Intron 1 |
|  | R:GGGATATTCTGGCCCAAAGTG |  |  | Intron 1 |
| STAT5A-Luc | F: TATGGTACCGAGGGAGGGAGGGGTCGA | 495 | 60 | Intron 1 |
|  | R: TAGCTCGAGCTGTGCGGGAAGAAAGATGGTGA |  |  | Intron 1 |
| STAT5A-Mut | F: AGAGCCGGAGAAGCAGACGC | - | - | - |
|  | R: GCGTCTGCTTCTCCGGCTCT | - | - | - |

**Table S2.** nsSNPs analyzed in porcine STAT5A with PhD-SNP, SIFT, SNAP, Meta-SNP and PolyPhen-2

| **SNPs** | **Substitution** | **PhD-SNP** | | **SIFT** | | **SNAP** | | | **Meta-SNP** | | | **Polyphen-2** | |
| --- | --- | --- | --- | --- | --- | --- | --- | --- | --- | --- | --- | --- | --- |
|  |  | **Prediction** | **Score** | **Prediction** | **Score** | | **Prediction** | **Score** | | **Prediction** | **Score** | **Prediction** | **Score** |
| c.561 T>A  rs1109669847 | Q52L | Neutral | 0.415 | Neutral | 0.260 | | Neutral | 0.475 | | Neutral | 0.240 | Neutral | 0.04 |
| c.870 G>T  rs1107818680 | T155K | Neutral | 0.402 | Neutral | 0.780 | | Neutral | 0.590 | | Neutral | 0.294 | Deleterious | 0.978 |
| c.872 G>T  rs1112766172 | Q156K | Neutral | 0.390 | Neutral | 0.900 | | Neutral | 0.395 | | Neutral | 0.206 | Neutral | 0.018 |
| c.877 G>T  rs1113275263 | D157E | Neutral | 0.100 | Neutral | 0.980 | | Neutral | 0.170 | | Neutral | 0.128 | Neutral | 0.002 |
| c.875 C>T  rs1111661398 | D157N | Neutral | 0.200 | Neutral | 0.570 | | Neutral | 0.215 | | Neutral | 0.239 | Neutral | 0.002 |
| c.879 G>T  rs1110630915 | T158K | Neutral | 0.210 | Neutral | 0.790 | | Neutral | 0.370 | | Neutral | 0.211 | Neutral | 0.417 |
| c.882 T>C  rs1108761552 | E159G | Neutral | 0.275 | Neutral | 0.340 | | Neutral | 0.400 | | Neutral | 0.186 | Neutral | 0.093 |
| c.881 C>T  rs1111465208 | E159K | Neutral | 0.397 | Neutral | 0.320 | | Neutral | 0.345 | | Neutral | 0.210 | Neutral | 0.054 |
| c.886 A>T  rs1108515671 | N160K | Neutral | 0.091 | Neutral | 0.880 | | Neutral | 0.210 | | Neutral | 0.136 | Neutral | 0.051 |
| c.887 C>T  rs1109301848 | E161K | Neutral | 0.101 | Neutral | 0.870 | | Neutral | 0.315 | | Neutral | 0.131 | Neutral | 0.001 |
| c.890 A>T  rs1108258192 | L162M | Neutral | 0.052 | Neutral | 0.190 | | Neutral | 0.305 | | Neutral | 0.103 | Neutral | 0.183 |
| c.1451 C>T  rs328250261 | A349T | Neutral | 0.049 | Neutral | 1.000 | | Neutral | 0.190 | | Neutral | 0.091 | Neutral | 0.086 |
| c.1581 G>C  rs321203224 | S392C | Neutral | 0.138 | Neutral | 0.230 | | Neutral | 0.335 | | Neutral | 0.163 | Neutral | 0.014 |
| c.2668 A>C  rs324086829 | D754E | Neutral | 0.298 | Neutral | 1.000 | | Neutral | 0.165 | | Neutral | 0.237 | Neutral | 0.003 |

**Table S3.** Putative transcriptional factor binding sites in the intron 1 of porcine STAT5A

| **SNP^a^** | **Variation** | **Gain^b^** | **Loss^c^** | **Score** | **Relative score** | **Prediction database** |
| --- | --- | --- | --- | --- | --- | --- |
| g.373 C>G | C>G | - | ZNF454 | 10.16 | 0.82 | JASPAR |
| g.508 A>C | A>C | - | E2F4 | 10.42 | 0.91 | JASPAR |
|  |  | - | TFDP1 | 8.75 | 0.86 |  |
| g.566 C>T | C>T | - | PRDM4 | 8.44 | 0.85 | JASPAR |
|  |  | RELB | - | 8.28 | 0.84 |  |
|  |  | Elk-1 | - |  |  | PROMO |

^a^ Based on position before exon 1; ^b,c^ Generated after substitution of allele 1 (wild type) with allele 2 (mutant).
